# Neuroimaging Findings in Pediatric Patients with Thalassemia Major

**DOI:** 10.3390/hematolrep14010009

**Published:** 2022-03-21

**Authors:** Yılmaz Akbaş, Sultan Aydın, Gökçen Öz Tunçer, Alper Köker, Yasemin Çoban, Gönül Oktay, Hakan Yeral

**Affiliations:** 1 Department of Pediatric Neurology, Mustafa Kemal University, Antakya 3106, Turkey; mberf@hotmail.com; 2 Department of Pediatric Hematology and Oncology, Hatay State Hospital, Antakya 3106, Turkey; 3 Department of Pediatrics, Hatay State Hospital, Antakya 3106, Turkey; gokcenoz@hotmail.com (G.Ö.T.); kokeralper@gmail.com (A.K.); yasemincoban83@gmail.com (Y.Ç.); 4 Department of Blood Disease Center, Hatay State Hospital, Antakya 3106, Turkey; drgonuloktay@hotmail.com; 5 Department of Radiology, Hatay State Hospital, Antakya 3106, Turkey; hakanyeral@hotmail.com

**Keywords:** thalassemia major, cranial MRI, neurological complications, hemoglobinopathy

## Abstract

Background: Cranial magnetic resonance imaging (MRI) studies about iron accumulation in children with thalassemia major are quite limited. Aim: This study aimed to detect neurological findings with cranial MRIs in the pediatric patients with thalassemia major who did not develop any neurological complications. Materials and Methods: Pediatric patients with thalassemia major who followed in the Pediatric Hematology Unit between 1 July 2017 and 1 January 2019 were included in the study. The patients underwent cranial MRI scans. Results: A total of 30 patients were included. The median age was 15 (range from 4–18) years old. We found that 7 patients had a splenectomy and 19 of the remaining 23 patients had splenomegaly. In addition, 13 of the patients had hepatomegaly, 10 had skeletal deformities, and 17 had growth retardation. The mean ferritin level was 3772.3 ± 2524.8. We detected various pathologies on cranial MRI images of 10 (33.3%) patients. In 3 of these patients, millimeter-sized ischemia-compatible lesions were found in the cerebral white matter, which did not fit any arterial area, and 5 patients had hyperintense lesions in the basal ganglia. Conclusion: Our study is valuable since 1/3 of our pediatric patients with thalassemia major were detected with intracranial pathology.

## 1. Introduction

Beta thalassemia is hypochromic microcytic anemia caused by a genetic mutation that leads to disorder in the production of the beta globulin chain in the structure of hemoglobin [1]. Although thalassemia can be seen worldwide, it is more common in East Asia, Middle East countries, Mediterranean countries, and North Africa. Every year, approximately 68,000 babies are born with thalassemia all over the world [2,3]. Thalassemia is divided into 3 groups as thalassemia major (TM), β-thalassemia intermedia, and β-thalassemia minor [4]. TM occurs as a result of a homozygous or compound heterozygous mutation in the beta globulin gene and constitutes the most severe picture that requires regular blood transfusions from the first year of life. Because regular blood transfusions are included in TM’s standard treatment, iron accumulation is the leading cause of complications. Iron chelators were used to prevent this. Frequent complications occur, especially after iron accumulation in cardiac and endocrine tissues. In addition, the neurological complications of iron accumulation may be followed up with noninvasive methods such as cranial magnetic resonance imaging (MRI) [5,6].

Like the cardiac and endocrine systems, the central nervous system is a tissue vulnerable to iron accumulation. Chronic ischemia, iron deposition, chelation-related neurotoxicity, and extramedullary hematopoiesis are the leading causes of neuronal tissue complications. It is necessary to identify these patients with neurophysiological and neuroimaging tests [7].

There are many studies showing iron accumulation in thalassemia major patients with cranial MRI. However, these studies were conducted either only in adults or inpatient groups with mixed adult and pediatric patients. Cranial MRI studies about iron accumulation in children with thalassemia major are quite limited. In this study, files for pediatric patients with thalassemia major who did not develop any neurological complications were scanned, and cranial MRIs were collected and the presence of cranial MRI findings was investigated.

## 2. Materials and Methods

This cross-sectional study was performed at the Pediatric Hematology Department between July 2017 and January 2019. Patients who have been followed with a diagnosis of transfusion-dependent thalassemia major (TM) aged 4 years and older were included in the study. The neurological examinations were performed for all patients to determine if neurological deficits were absent or present. There were formal assessments made of cognitive function. Those with normal school achievement were included. Informed consent was obtained from all participants. The patients with cranial MRI images obtained by scanning these patients’ files were included in the study. Demographic data, physical examination findings of the patients, age at diagnosis, follow-up period, chelation therapy, splenectomy history, and annual blood transfusion amount (units/year) were obtained from the file. Pre-transfusion hemoglobin (Hb) and serum ferritin levels in the recent admission were measured for each patient. Cranial MRI studies were achieved. Patients with abnormal neurological examinations, any known central nervous system disease, secondary hematological disease, older than 18 years, and patients who were followed for less than 1 year were excluded.

## 3. Cranial Magnetic Resonance Imaging

All cranial MRI examinations were performed using 1.5-Tesla images (Symphony, Siemens, Erlangen, Germany). T1 (TR/TE: 450/13 ms; slice thickness: 3 mm; matrix size: 256_256; field of view (FOV) 20 cm) and T2 (TR: 2000 ms; TE: 15–120 ms; slice thickness: 3 mm; matrix size: 256_256; FOV: 20 cm) sequences were shot in coronal sagittal and axial sections.

## 4. Statistical Analysis

SPSS, the Statistical Package for the Social Sciences (SPSS) software, version 21 (IBM Corporation, Armonk, NY, USA), was used for the analyses. Descriptive statistics were used. Data (%) are summarized as median and mean ± standard deviation. A Chi-square test was used to investigate whether there was any dependency between variables. The Mann–Whitney U test was used to compare nonparametric variables, and *p* < 0.05 was considered statistically significant.

## 5. Results

A total of 30 patients, 18 male (60%) and 12 (40%) female, aged between 4 and 18 years (mean age 13.96 ± 3.1), were included. Nineteen (63.3%) of the patients were Turkish, and 11 (36.7%) were Syrian children who fled the civil war.

The age of diagnosis was available only for 20 patients, and these patients were diagnosed at an average age of 10 months (range from 4–84 months). All patients, except one, started receiving regular erythrocyte transfusions from the time of their diagnosis. One remaining patient started to receive transfusions 29 months after the diagnosis. The amount of erythrocyte transfusion for patients was determined as 25.9 ± 8.3 (mean ± SD) units/year. Twenty patients were receiving regular iron chelation therapy. It was found that the patients received an average of 10.5 ± 3.3 years of chelation therapy. To examine the effectiveness of chelation therapy, pre-chelation ferritin levels and final ferritin levels were compared. Although the patients received chelation therapy, there was no statistically significant change in ferritin levels (Table 1).

Considering the physical examination findings, we found that 7 patients had a splenectomy and 19 of the remaining 23 patients had splenomegaly. In addition, 13 of the patients had hepatomegaly, 10 had skeletal deformities, and 17 had growth retardation. No statistically significant difference was found between the physical examination findings and gender. However, when the physical examination findings of Turkish and Syrian patients were compared, skeletal deformities were found to be significantly higher in Syrian patients (*p* = 0.001) (Table 2).

**Figure 1:**
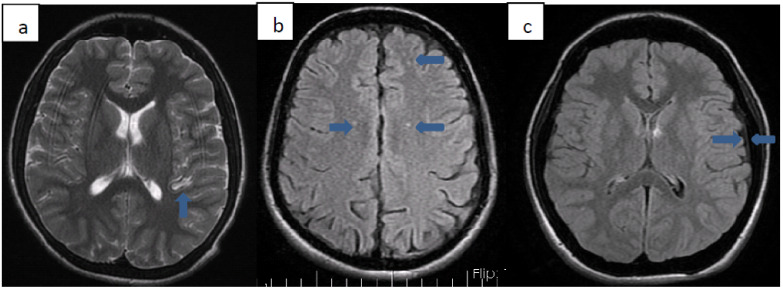
(**a**–**c**): Axial T2 (**a**) and Axial FLAIR (**b**) weighted images show multiple foci of hyperintensity that are related to asymptomatic brain ischemia. Axial FLAIR (**c**) image show widened epidural tissue.

We detected various pathologies on cranial MRI images of 10 (33.3%) patients. Patients with pathology on MRI are summarized in Table 3. Three of these patients (33.3%) were Syrian citizens. The average age of diagnosis of patients with pathology on MRI was 25 months. All but one of the patients received transfusion therapy from the time of diagnosis. Transfusion treatment was started 29 months after diagnosis, only for patient number 9. The total chelation time of the patients except 3 was determined. Accordingly, the average duration of chelation therapy these patients received was 9.7 years. In 3 of these patients, millimeter-sized ischemia-compatible lesions were found in the cerebral white matter, which did not fit any arterial area. Five patients had hyperintense lesions in the basal ganglia (2 globus pallidus, three putamens). One of our patients had thickening of the dura mater, and one of our patients had cerebral atrophy. Cardiac involvement was not found in any of these patients. While only patient 4 had insulin resistance, the other patients did not have any endocrinopathy. No significant difference was found between gender or nationality and MRI pathologies (Figure 1).

Characteristics of patients with and without pathology on MRI were compared (Table 4). When the age of diagnosis of both groups, ferritin level before and after chelation treatment, the average amount of transfusion, the duration of chelation treatment, Hb, Plt, Wbc values was compared, no statistically significant difference was found. There was also no difference between these two groups in terms of gender (*p* = 0.65) and nationality (*p* = 0.45).

## 6. Discussion

Thalassemia major is a condition that leads to the need for regular blood transfusions from the first year of life. Although chelation therapy is given for the accumulated iron, iron accumulation may occur in various tissues and cause dysfunction in organs. In particular, cardiac and endocrine problems frequently occur in these patients. Another tissue at risk is the nervous system. Common neurological complications are seen due to hypoxia, iron accumulation, chelation-related neurotoxicity, and extramedullary hematopoiesis [8,9,10].

In our study, we found various pathologies on the cranial imaging of 1/3 of our patients. Five of these had hyperintense lesions secondary to iron accumulation in the basal ganglia. Three of the remaining patients had millimetric ischemic foci due to hypoxia, 1 had dura mater thickening due to extramedullary hematopoiesis, and 1 had cerebral atrophy. The most common pathology we encountered in cranial MRIs was lesions in the basal ganglia. Three of our patients had lesions in the putamen and 2 in the globus pallidus. Metafratzi et al. showed that more iron accumulates in the putamen caudate nucleus and cortex in patients with thalassemia major than in healthy people. In various studies performed later, it was shown that iron accumulation is also present in the thalamus, red nucleus, and choroid plexus [9,10,11,12]. The second most common cranial MRI pathology was millimetric ischemic changes. We found ischemic foci in the cerebral white matter in 3 of our patients. In recent studies, thromboembolic events were reported with a rate of 0.9–29% in thalassemia patients [13,14,15,16,17,18,19]. These ischemic foci that we saw in our patients were minimal areas due to microemboli. Dural thickening due to extramedullary hematopoiesis is extremely rare. Studies of dural thickening resulting from intracranial extramedullary hematopoiesis in children with thalassemia are generally at the level of case reports [18,19]. In our study, we detected dural thickening in 1 patient. While extramedullary hematopoiesis is more common in other parts of the body, it is rare intracranially.

When those with and without pathology on MRI were compared in terms of pre- and post-chelation ferritin levels, duration of chelation therapy, and age at diagnosis, there was no significant difference between the two groups in our study. Serum ferritin cannot pass the blood-brain barrier, but transferrin-bound iron turns into hemosiderin in neurons, and if this process is excessive, iron accumulation occurs in the brain [20,21]. In a study conducted by Akhlaghpoor et al. in 2012 in adult TM patients, no relationship was found between serum ferritin level and iron accumulation in cranial MRI [9].

Because Syrian patients have poor nutritional conditions, receiving irregular erythrocyte suspension, and inadequate and inappropriate use of chelation, the skeletal deformity in Syrian patients was significantly higher than in Turkish patients (*p*: 0.001). In addition, they had hyperferritinemia.

In conclusion, our study is valuable since it only includes pediatric patients with thalassemia major. Detection of intracranial pathology in 1/3 of our patients is one of the valuable results of our study. None of our patients with intracranial pathology had any neurological complications. 

## Figures and Tables

**Table 1:** Comparison of ferritin levels before chelation therapy with final ferritin level.

		Before Chelation Therapy	Final	*p*
Ferritin	Mean	3772.3	3899.4	0.794
	Median	3133	2979	
	SD	2524.8	3007.2	

SD: Standard deviation.

**Table 2:** Comparison of physical examination findings of Turkish and Syrian patients.

	Turkish		Syrian		*p*
	Yes	No	Yes	No	
Growth retardation	9	10	8	3	0.167
Splenomegaly	12	7	7	4	0.646
Hepatomegaly	7	12	6	5	0.287
Skeletal deformities	2	17	8	3	**0.001**

**Table 3:** Characteristics of patients with pathology on MRI.

Patient	Age	Nationality	Sex	Age at Diagnosis (Month)	Chelation	Duration of Chelation (Year)	Hepatosplenomegaly	Splenectomy	CNS
1	13	T	M	3	deferasirox	11	-	+	White matter
2	10	T	M	36	deferasirox + deferiprone	5	+	-	Putamen
3	16	T	M	12	deferasirox	11	-	-	White matter
4	18	T	M	48	deferasirox	11	-	-	Thalamus
5	15	T	F	36	deferasirox	6	+	-	Duramater
6	17	T	M	9	deferasirox + deferiprone	15	+	-	Duramater
7	11	S	F	n/a	deferasirox	n/a	-	+	Globus pallidus, Putamen
8	16	S	M	n/a	deferasirox + deferiprone	n/a	-	+	White matter
9	15	T	F	31	deferasirox	9	+	-	Cortical atrophy
10	10	S	F	n/a	deferasirox + deferiprone	n/a	+	-	Globus pallidus, Putamen

T = Turkish, S = Syrian, M = Male, F = Female.

**Table 4:** Comparison of patients with and without pathology on MRI.

	MRI Pathology	n	Mean + SD	*p*
Age at diagnosis	Yes	7	25.0 + 16.9	0.41
	No	13	16.9 + 22.0	
Hb	Yes	10	9.4 + 1.02	0.42
	No	20	9.0 + 1.00	
Plt	Yes	10	447.5 + 208.5	0.89
	No	20	434.7 + 286.4	
Wbc	Yes	10	14.8 + 9.8	0.76
	No	20	15.8 + 7.9	
Amount of ES transfusion	Yes	10	27.9 + 8.02	0.28
	No	20	24.3 + 9.05	
Duration of Chelation	Yes	7	9.7 + 3.4	0.97
	No	13	9.7 + 4.3	
Before therapy Ferritin	Yes	9	3832.6 + 3146.2	0.93
	No	12	3727.0 + 2094.3	
After therapy Ferritin	Yes	10	3057.9 + 2125.3	0.35
	No	20	3952.4 + 3002.9	

Hb: Hemoglobin, Plt: Platelet, Wbc: White blood cell, ES: erythrocyte suspension, SD: Standard deviation, MRI: Magnetic resonance imaging.

## Data Availability

Datasets can find in section “MDPI Research Data Policies” at https://www.mdpi.com/ethics (accessed on 14 February 2022).

## References

[1] Rund D., Rachmilewitz E. (2005). Beta-thalassemia. N. Engl. J. Med..

[2] Weatherall D.J., Williams T.N., Allen S.J., O’Donnell A. (2010). The population genetics and dynamics of the thalassemias. Hematol. Oncol. Clin. N. Am..

[3] Cao A., Galanello R. (2010). Beta-thalassemia. Genet. Med..

[4] Modell B., Darlison M. (2008). Global epidemiology of hemoglobin disorders and derived service indicators. Bull. World Health Organ..

[5] St Pierre T.G., Clark P.R., Chua-anusorn W., Fleming A.J., Jeffrey G.P., Olynyk J.K., Pootrakul P., Robins E., Lindeman R. (2005). Non-invasive measurement and imaging of liver iron concentrations using proton magnetic resonance. Blood.

[6] Anderson L.J., Holden S., Davis B., Prescott E., Charrier C.C., Bunce N.H., Firmin D.N., Wonke B., Porter J., Walker J.M. (2001). Cardiovascular T2-star (T2*) magnetic resonance for the early diagnosis of myocardial iron overload. Eur. Heart J..

[7] Nemtsas P., Arnaoutoglou M., Perifanis V., Koutsouraki E., Orologas A. (2015). Neurological complications of beta-thalassemia. Ann. Hematol..

[8] Metafratzi Z., Argyropoulou M.I., Kiortsis D.N., Tsampoulas C., Chaliassos N., Efremidis S.C. (2001). T2 relaxation rate of basal ganglia and cortex in patients with b-thalassaemia major. Br. J. Radiol..

[9] Akhlaghpoor S., Ghahari A., Morteza A., Khalilzadeh O., Shakourirad A., Alinaghizadeh M.R. (2012). Quantitative T2* magnetic resonance imaging for evaluation of iron deposition in the brain of β-thalassemia patients. Clin. Neuroradiol..

[10] Qiu D., Chan G.C., Chu J., Chan Q., Ha S.Y., Moseley M.E., Khong P.L. (2014). MR quantitative susceptibility imaging for the evaluation of iron loading in the brains of patients with NL -Thalassemia major. AJNR Am. J. Neuroradiol..

[11] Hasiloglu Z.I., Asik M., Ure E., Ertem F., Apak H., Albayram S. (2017). The utility of susceptibility-weighted imaging to evaluate the extent of iron accumulation in the choroid plexus of patients with β-thalassaemia. Clin. Radiol..

[12] Manara R., Ponticorvo S., Tartaglione I., Femina G., Elefante A., Russo C., Carafa P.A., Cirillo M., Casale M., Ciancio A. (2019). Brain iron content in systemic iron overload: A beta-thalassemia quantitative MRI study. Neuroimage Clin..

[13] Wong V., Li A., Lee A.C. (1993). Neurophysiologic study of beta thalassemia patients. J. Child Neurol..

[14] Cappellini M.D., Robbiolo L., Bottasso B.M., Coppola R., Fiorelli G., Mannucciet A.P. (2000). Venous thromboembolism and hypercoagulability in splenectomized patients with thalassaemia intermedia. Br. J. Haematol..

[15] Eldor A., Durst R., Hy-Am E., Goldfarb A., Gillis S., Rachmilewitz E.A., Abramov A., MacLouf J., Godefray Y.C., De Raucourt E. (1999). A chronic hypercoagulable state in patients with beta-thalassaemia major is already present in childhood. Br. J. Haematol..

[16] Michaeli J., Mittelman M., Grisaru D., Rachmilewitz E.A. (1992). Thromboembolic complications in beta thalassemia major. Acta Haematol..

[17] Haghpanah S., Karimi M. (2012). Cerebral thrombosis in patients with beta-thalassemia: A systematic review. Blood Coagul. Fibrinolysis.

[18] Merchant R., Choudhari A.J., Verma M., Patkar D.P., Doctor P. (2018). Intracranial Hematopoiesis in Beta Thalassemia: A Case Series. Indian J. Pediatr..

[19] Karki B., Xu Y.K., Tamrakar K., Wu Y.-K. (2012). Intracranial extramedullary hematopoiesis in beta-thalassemia. Korean J. Radiol..

[20] Van Gelder W., Huijskes-Heins M.I., Cleton-Soeteman M.I., van Dijk J.P., van Eijk H.G. (1998). Iron uptake in blood-brain barrier endothelial cells cultured in iron-depleted and iron-enriched media. J. Neurochem..

[21] Bizzi A., Brooks R.A., Brunetti A., Hill J.M., Alger J.R., Miletich R.S., Francavilla T.L., Di Chiro G. (1990). Role of iron and ferritin in MR imaging of the brain: A study in primates at different field strengths. Radiology.

